# Accelerated Apixaban Removal by Using the ADVanced Organ Support (ADVOS) Albumin Hemodialysis System—A Case Report

**DOI:** 10.1055/a-2682-8640

**Published:** 2025-09-15

**Authors:** Bernd Panholzer, Ulrike Nowak-Goettl, Katharina Huenges, Wiebke Sommer

**Affiliations:** 1 Department of Cardiac Surgery, University Hospital Schleswig-Holstein, Kiel, Germany; 2 Institute of Clinical Chemistry, University Hospital Schleswig-Holstein, Kiel, Schleswig-Holstein, Germany

**Keywords:** cardiogenic shock, direct oral anticoagulants, cardiac surgery, extracorporeal organ support

## Abstract

**Background:**

In patients on direct oral anticoagulants (DOAC), emergency surgery is characterized by the occurrence of a massively increased tendency to bleed. Currently, there is no approved antidote for postoperative patients, making specific therapy challenging in these situations.

**Case Description:**

Emergency surgery was required for a 72-year-old male patient who was in cardiogenic shock due to severe aortic regurgitation resulting from acute prosthetic valve endocarditis. Due to atrial fibrillation, the patient was on apixaban, a factor Xa (FXa) inhibitor anticoagulant, until surgery. We used the ADVanced Organ Support (ADVOS) albumin hemodialysis system postoperatively to treat persisting shock with multi-organ failure, acidosis, and DOAC removal. Serial drug-level measurements revealed strongly accelerated apixaban clearance. In line with this, we observed only moderate drainage losses.

**Conclusion:**

ADVOS accelerates the removal of apixaban and is a promising therapy for preventing bleeding complications in patients receiving DOAC therapy after emergency surgery.

## Background

Infectious endocarditis (IE) is a life-threatening and highly complex multisystem disease of cardiovascular structures, caused by different pathogens. Antimicrobial therapy is the cornerstone in the treatment of IE, but in up to 50% of patients, treatment escalation by surgical intervention is required. 1 The three main reasons for patients with acute IE to undergo surgery are cardiac failure, septic embolization prevention, and uncontrolled infection. Depending on the severity of the symptoms mentioned, the recommended timing for performing the surgery varies: in non-urgent cases, it should be performed within the same hospital admission; in urgent cases, between 3 and 5 days; and a 24-hour time frame is recommended in emergency cases. 2 Leaflet perforation or rupture unavoidably leads to new severe valvular regurgitation or worsening of pre-existing valvular regurgitation. Subsequently, patients develop acute or acute on chronic heart failure (HF), which culminates in cardiogenic shock in up to 5% of cases. This acute life-threatening condition with progressive hemodynamic deterioration, pulmonary edema, and multi-organ dysfunction does not allow any postponement of surgery, since there is no other treatment option in these situations. Prosthetic valve endocarditis (PVE), i.e., the infection of cardiac prosthetic material, occurs in 1 to 6% of patients with valve prostheses and accounts for 20 to 30% of all cases of IE. Compared with native valve endocarditis (NVE), the severity of PVE is increased with increased in-hospital mortality of up to 20 to 40% 3 as well as reduced long-term survival. 4 Furthermore, early PVE, defined as the infection of prosthetic valves within the first year after valve replacement, is considered to be a separate entity associated with even higher mortality rates, 5 and conservative treatment with antibiotics is unlikely to lead to a cure. Unfortunately, early repeat surgery, with its increased complexity due to severe adhesions of the tissue and the higher incidence of perivalvular complications such as abscess or dehiscence of prosthesis due to annular tissue destruction, is challenging and associated with a high rate of complications, such as bleeding.

Surgical procedures for patients on direct oral anticoagulants (DOAC) are associated with a massively increased tendency to bleed. Hence, guidelines strongly recommend cessation of these drugs for an appropriate time, usually 2 to 7 days, before surgery. 6 Depending on the specific substance, and gastrointestinal and liver function, the effect duration remains up to several days. Apixaban (Eliquis®, Bristol-Myers Squibb GmbH & Co. KGaA/Pfizer Pharma GmbH) is a factor Xa (FXa) inhibitor anticoagulant indicated to reduce the risk of stroke and systemic embolism in patients with nonvalvular atrial fibrillation and for prophylaxis and treatment of deep vein thrombosis and pulmonary embolism. Due to its long half-life of 12 hours during repeat dosing, the manufacturer recommends discontinuation of the medication at least 48 hours before surgery. Plasma protein binding of apixaban in humans is approximately 87%, and its volume of distribution is approximately 21 L. Both urine and feces eliminate apixaban, with renal excretion accounting for about 27% of the total clearance. Biliary and direct intestinal excretion contribute to the elimination of apixaban in the feces. 7

We hypothesize that the use of blood purification aiming to remove protein-bound DOAC both peri- and postoperatively may aid in regaining hemodynamic stability and improving the prognosis in patients requiring emergency surgery due to infectious endocarditis.

## Case Description

**Fig. 1:**
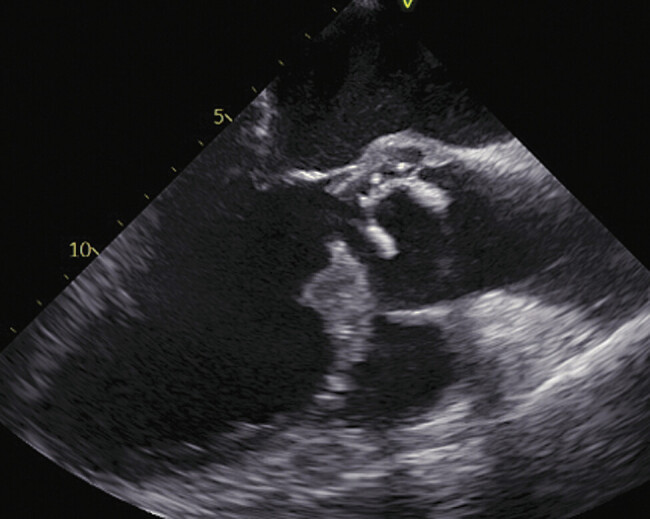
Prosthetic valve endocarditis.

Our emergency department admitted a 73-year-old male patient with a history of prior aortic valve replacement due to acute chest pain, dyspnea, and cough. Upon admission, echocardiography revealed a severe destruction of the aortic valve prosthesis. Severe paravalvular aortic regurgitation and strongly increased prosthesis mobility suggested an acute infection of the aortic annulus that had already started tearing out of the valve ( Fig. 1 ). The patient had been taking apixaban due to atrial fibrillation until the morning of the day of admission. In line with this, the apixaban levels obtained in the emergency department were increased (101 ng/mL) up to the therapeutic range. Considering the recent surgery 3 months ago, the massively increased tendency to bleed after DOAC intake, and the hemodynamic stability of the patient in the initial phase, the patient was transferred to our cardiosurgical ICU for conservative treatment until the end of the apixaban effect. Directly after ICU admission, the hemodynamics of the patient rapidly deteriorated. Moreover, he developed pulmonary edema and respiratory failure, conditions that could not be controlled by noninvasive ventilation. The severe aortic valve regurgitation and the severe, septic-induced vasoplegia led to a rapidly progressive shock event, despite the echocardiographically preserved biventricular function. In the absence of any sensible therapeutic options, an indication for emergency surgery as a rescue procedure was made.

**Fig. 2:**
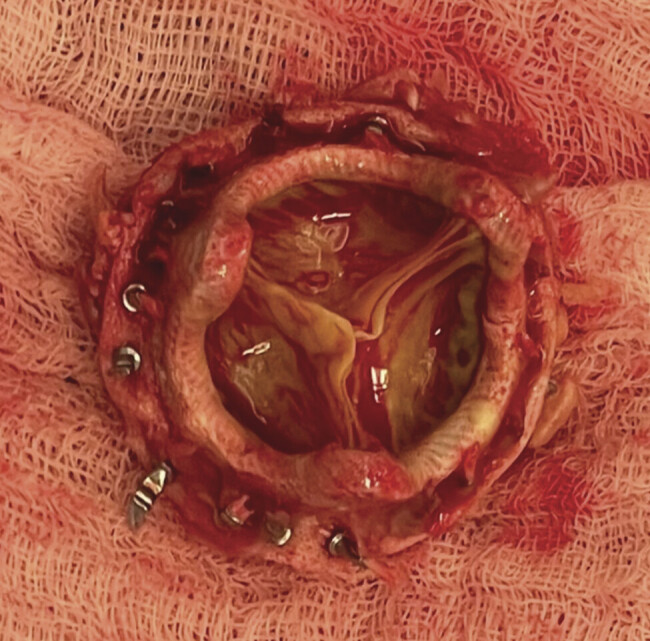
Aortic valve prosthesis with a loss of nearly the complete valve fixation.

Due to the therapeutic levels of apixaban, cytokine adsorption (Cytosorb®, CytoSorbents Europe GmbH, Berlin, Germany) for apixaban removal was used during cardio-pulmonary bypass (CBP). Intraoperatively, we saw a massive infection of the aortic valve prosthesis and the annulus, with a loss of nearly the complete valve fixation ( Figs. 1 and 2 ). As the annulus was destroyed by the infection, extensive debridement was necessary, followed by annulus reconstruction with bovine pericardium and re-implantation of a biological aortic valve (27 mm Magna Ease, Edwards Lifesciences Corp., Irvine, Canada). During surgery, a point-of-care coagulation examination guided the administration of prothrombin complex concentrate (PCC), fibrinogen, and fresh-frozen plasma, followed by heparin antagonization.

Despite a successful valve replacement, the deterioration of the patient's hemodynamics could not be reversed. Echocardiography revealed a severely impaired biventricular function. Furthermore, the patient developed vasoplegic shock and a metabolic disorder with lactate acidosis. To avoid any potential affection of the recently implanted aortic valve, implantation of a microaxial pump was not performed, but extracorporeal life support (ECLS) via femoro-femoral access was implanted. Lactate (16.7 mmol/L) and vasoactive inotropic score (152) were strongly elevated at ECLS implantation. Given the severity of the cardiogenic shock and the significantly elevated oxygen deficit, we sought the highest possible circulatory support and oxygenation, establishing a flow of 4.8 L/min at 100% FiO _2_ . We maintained these parameters until we achieved hemodynamic and metabolic stabilization, at which point we considered the start of ECLS weaning possible. For the treatment of exaggerated inflammation, metabolic acidosis (pH 7.17), and acute renal failure, an ADVanced Organ Support albumin hemodialysis system (ADVOS multi, ADVITOS GmbH, Munich, Germany) was established. At the beginning, settings for ADVOS treatment were a blood flow rate of 150 mL/min and a concentrate flow of 160 mL/min. Dialysate pH was 7.8, and because the patient was highly volume-responsive, the ultrafiltration rate was 0 mL/h initially. Considering the documented efficient removal of protein-bound substances, 8 we hypothesized that this would accelerate apixaban removal.

**Fig. 3:**
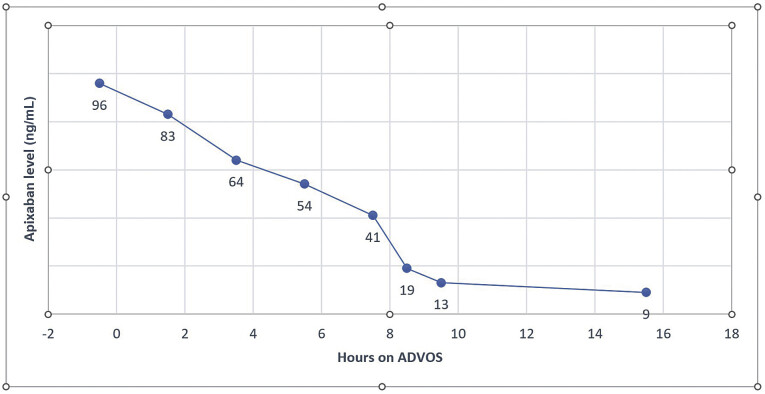
Kinetics of the accelerated decrease of the apixaban levels.

Apixaban levels were measured after surgery, shortly before the start, and during ADVOS at narrow time intervals with a multiparameter coagulation analyzer equipped with a photo-optical clot-detection unit (ACLTOP 750, Werfen GmbH, Munich, Germany). These showed a rapid and, compared with the known kinetics, strongly accelerated decrease of the apixaban levels ( Fig. 3 ). At ICU admission and before the start of ADVOS, the initial apixaban value was 96 ng/mL. However, after 5 hours of ADVOS therapy, it dropped to 54 ng/mL, and 3 hours later, we measured levels of 19 ng/mL. Our institutional protocol assumes that apixaban levels below 30 ng/mL are safe in terms of bleeding risk. Consistent with this, we observed a moderate drainage loss of 800 mL/24 hour, leading to the patient's stabilization. It was necessary to transfuse three packages of red blood cells, one package of platelets, and four packages of fresh-frozen plasma in the first 24 hours after postoperative ICU admission and the start of ADVOS. Furthermore, the metabolic acidosis documented before the start of therapy completely disappeared within a short period of time (pH 7.35 after 90 minutes of treatment).

Even though hemodynamics improved, and right ventricular function recovered, we noticed persistently severe impaired left ventricular function in the following days, so an Impella 5.5 (Abiomed Europe GmbH, Aachen, Germany) was implanted via the left axillary artery. Under this therapy, the patient's overall condition continued to improve significantly. ECLS was explanted on the 4th postoperative day, followed by Impella explantation on day 7. After cardiopulmonary decompensation with pulmonary edema, our patient developed pneumonia and a tracheotomy was necessary. The patient was discharged to a weaning rehabilitation center 22 days after emergency surgery for PVE. Decannulation became possible 12 days after his transfer to the external weaning clinic.

## Discussion

This is the first report of a rapid and effective reduction of apixaban plasma levels using albumin-based hemodialysis (ADVOS), to the best of our knowledge. In the present case, we report on a patient requiring emergency cardiac surgery while on DOAC therapy. Apixaban, a direct FXa inhibitor, has received approval for multiple indications and is currently in widespread use. Thus, its removal perioperatively and postoperatively is of the utmost importance to avoid bleeding complications. Despite the potential use of several strategies, their effectiveness in emergency surgery situations remains incompletely understood. In Germany, andexanet alfa (Ondexxya, AstraZeneca AB, Södertälje, Sweden) is approved to reverse anticoagulation in cases of life-threatening or uncontrolled bleeding in adult patients treated with a direct FXa inhibitor such as apixaban or rivaroxaban. However, experience with surgical patients is very limited. ANNEXA-4, 9 a multicenter, prospective, open-label, single-group study, showed substantially reduced anti–factor Xa activity after andexanet alfa infusion in nonsurgical patients. Moreover, international guidelines do not approve or recommend its use for emergency surgical patients, making it considered off-label. In addition, a higher incidence of thromboembolic complications has been described, as well as an altered heparin effect, 10 leading to clot formation in the extracorporeal circulation. This unfavorable risk–benefit profile in our postoperative patient on ECLS, as well as our own experience, prevented us from using this medication.

A successful apixaban reduction by using cytokine adsorption (Cytosorb) has already been described several times in the literature. 11 12 Hence, we used this intraoperatively during the phase on CBP. However, the period for this application was relatively short, so it cannot be assumed that a sufficient amount of apixaban was removed. Elevation of the apixaban levels during the first measurement in the ICU confirmed this fact.

The test we used is accredited in Germany and is based on chromogenic and immunological assays. It follows a two-step principle of inhibition and cleavage of added FXa and subsequent photometric concentration determination. Disturbing factors such as hypertriglyceridemia, hyperbilirubinemia, or increased hemoglobin concentrations have no influence at low concentrations. A residual heparin effect can be ruled out by the fact that it was fully antagonized by protamine. Indeed, the last administration of unfractionated heparin (UFH) intraoperatively was more than 5 hours before measurement, and activated clotting time was postoperatively below 120 seconds in serial measurements.

Renal excretion plays a minor role in the degradation of apixaban; hence, standard renal replacement therapy is not a sufficient method to eliminate apixaban. Since our patient was in severe shock with vasoplegia due to excessive immune reaction and severe acidosis (pH 7.17), we decided to start a multi-organ support with the ADVOS. The ADVOS procedure combines a standard high-flow dialysis with an albumin dialysis for improved removal of protein-bound toxins. The core process involves recycling of toxin-loaded human serum albumin dialysate by altering the pH and temperature in the regeneration circuit to reuse the dialysate. This is done by transferring the dialysate to an acid leg and a base leg. This in turn allows the user to adjust the pH value of the dialysate, which enables a very quick and complete compensation of acidosis. 13 Compared with standard therapy (such as buffering with sodium bicarbonate), the effect of ADVOS on acidosis is advantageous for several reasons: The so-called ADVOS Multi Circuit allows for individual pH value selection of up to 9.5, ensuring rapid and precise acidosis compensation. Due to the existing degree of concentration between the blood and ADVOS multi-circulation, the H ^+^ ions pass into the latter. Based on this principle, there is no conversion of H ^+^ ions and bicarbonate to CO _2_ and H _2_ O; therefore, there is no risk of CO _2_ retention or respiratory acidosis. In addition, there is no volume or sodium load. Plasma protein binding of apixaban in humans is high, and detoxification of protein-bound toxins as well as elimination of protein-bound drugs 14 through ADVOS is proven, 7 so the accelerated removal of apixaban we measured was expected. We hypothesize that the efficient removal of apixaban together with the rapid correction of acidosis helped to restore hemodynamic stability, as previously reported. 15 Besides this, no device-related adverse events were reported during the treatment. One ADVOS application, which lasts for 24 hours, costs around 2,500 euros. Hence, it can be considered far more cost-effective when compared with the off-label antidote.

The present case demonstrates that advanced albumin-based dialysis, such as ADVOS, can significantly accelerate apixaban removal, enabling major cardiac surgery with low postoperative bleeding. Hence, it could be a promising strategy to avoid bleeding in postoperative patients under apixaban medication.
